# Supplementary material for: Improving the Assessment Process of Family Functioning in Adult Bipolar Disorders: A PRISMA Systematic Review
Source: J Clin Med. 2022 Feb 5;11(3):841. doi: 10.3390/jcm11030841 (PMC8836941; doi:10.3390/jcm11030841)
Supplement: Supplementary file 1 [file jcm-11-00841-s001.zip › jcm-1470739-SI.pdf]

Data S1. Eligibility criteria

	Inclusion criteria	*Exclusion criteria*
Language	English or French	Any other language
Publication date	No restriction	No restriction
Publication status	Published original research study	Review articles, books, book chapters, theses, editorials, guidelines, conference abstracts, indexes and model proposals
Types of participants	Adults diagnosed with BD	Participants under 18 years old<br>Participants without BD
Types of outcome measures	FF concept assessed in the current family	Other concepts than FF (such as parental and marital relationships)<br>FF of the family-of-origin

Note: BD = Bipolar Disorders; FF = family functioning

Data S2. *Example of Electronic Search Strategy*

<u>Search in PubMed:</u>

Search: **"family functioning" AND "bipolar disorder"**

"family functioning"[All Fields] AND "bipolar disorder"[All Fields]
